# High-Grade Inflammation Attenuates Chemosensitivity and Confers to Poor Survival of Surgical Stage III CRC Patients

**DOI:** 10.3389/fonc.2021.580455

**Published:** 2021-04-23

**Authors:** Hou-Qun Ying, Xia-Hong You, Yu-Cui Liao, Fan Sun, Xue-Xin Cheng

**Affiliations:** ^1^Department of Nuclear Medicine, Jiangxi Province Key Laboratory of Laboratory Medicine, The Second Affiliated Hospital of Nanchang University, Nanchang, China; ^2^Institute of Translational Medicine, Zhejiang University School of Medicine, Hangzhou, China; ^3^School of Public Health, Nanchang University, Nanchang, China; ^4^Jiangxi Provincial Key Laboratory of Preventive Medicine, Nanchang University, Nanchang, China; ^5^Department of Clinical Laboratory, Jiangxi Province Key Laboratory of Laboratory Medicine, The Second Affiliated Hospital of Nanchang University, Nanchang, China; ^6^Biological Resource Center, The Second Affiliated Hospital of Nanchang University, Nanchang, China

**Keywords:** primary tumor location, chronic inflammation, FPR, colorectal cancer, chemosensitivity

## Abstract

**Background:** Heterogeneous clinical and molecular characteristics are reported in colorectal cancer (CRC) with different tumor laterality. However, the outcome of left- and right-sided patients with stage I–III CRC and the role of chronic inflammation in survival differences between them remain unclear.

**Method:** A prospective study including 1,181 surgical patients with stage I–III CRC was carried out to investigate the involvement of circulating fibrinogen-to-pre-albumin (Alb) ratio (FPR) and primary tumor sidedness in the clinical outcome of those patients. We further investigated the effect of FPR on adjuvant chemotherapy response and recurrence in stage III patients.

**Results:** Our study showed that the right tumor location was significantly associated with poor recurrence-free survival (RFS) (*p* = 0.04, adjusted HR = 1.41, 95% CI = 1.02–1.94) and overall survival (OS) (*p* = 0.04, adjusted HR = 1.55, 95% CI = 1.01–2.38) only in the stage III disease. In these patients, T4 stage distribution (83.39 vs. 70.94%, *p* < 0.01) within right-sided cases was significantly higher than left-sided patients. Moreover, preoperative FPR within right-sidedness (*p* < 0.01), T4 stage (*p* < 0.05), and large cancer bulk (≥5 cm) (*p* < 0.05) subgroups was significantly elevated compared to their counterparts, and it was gradually rising following the increased cancer bulk (*p* trend < 0.01). High-FPR distribution (52.30 vs. 27.00%, *p* < 0.01) within right-sided patients with the stage III disease was significantly higher than that in the left-sided cases. RFS (*p*_log−rank_ < 0.01) and OS (*p*_log−rank_ < 0.01) of the high-FPR patients were extremely inferior to the low-FPR cases, and the significant associations were observed when they were adjusted by other confounders including primary tumor location (*p* < 0.01, adjusted HR = 1.96, 95% CI = 1.42–2.70 for RFS; *p* < 0.01, adjusted HR = 2.44, 95% CI = 1.59–3.75 for OS). Additionally, RFS of adjuvant chemotherapy-treated high-FPR patients was superior to the patients without chemotherapy (*p*_log−rank_ = 0.01) but was inferior to the low-FPR patients undergoing the treatment, especially in the 5-FU- and XELOX-treated subgroup.

**Conclusion:** These findings indicate that chronic high-grade inflammation weakens chemotherapy efficacy and contributes to the poor prognosis of stage III surgical CRC patients.

## Introduction

Colorectal cancer (CRC) is a kind of heterogeneous malignancy with different clinical options and diverse therapeutic outcome (1, 2). Recently accumulated evidence shows that proximal colon cancer and the distal disease including rectal cancer harbor strikingly distinct clinical characteristics and immune and molecular profiles (3–6). Thus, the left-sided (distal colon and rectal cancer) and right-sided (proximal colon cancer) diseases are considered as two distinct entities (7, 8).

A large amount of data consistently shows that metastatic CRC (mCRC) cases with left-sidedness derive meaningful benefit from anti-epidermal growth factor receptor antibody (9–11). Our previous studies also imply that the prognosis of bevacizumab-treated left-sided mCRC patients is better than the counterpart (12, 13). These findings reveal that tumor laterality can affect and predict the efficacy of target therapy within the advanced disease. Meanwhile, several large-scale cohort studies investigated the effect of tumor sidedness on early-stage patients with the treatment of standard chemotherapy (14–18). Unfortunately, no consistent conclusion is achieved and the debate continues. Karim et al. reported no overall survival (OS) and cancer-specific survival (CSS) difference between the right- and left-sided patients with stage I–III or III colon cancer (15). On the contrary, a poor prognosis in terms of recurrence-free survival (RFS) and OS was reported in stage III right-sided colon cancer patients undergoing adjuvant chemotherapy (14, 17). More interestingly, a recent study performed by Ishihara et al. observed a low recurrence rate in stage II–III patients with right-sidedness compared to the left-sided cases; however, 5-year CSS within the left-sided patients was significantly longer than those with the right-sided disease (18).

Chronic inflammation is one hallmark of malignancies including CRC (19, 20). The inflammatory processes can regulate low expressions of circulating albumin (Alb) and pre-Alb and up-regulated plasma fibrinogen (Fib). Our previous study showed that circulating Fib-to-pre-Alb ratio (FPR) could sensitively imply the body response to chronic inflammation in solid malignancies (21), including hepatocellular carcinoma (22), and gastric cancer (23) as well as CRC (24), and it was superior to other inflammatory ratios or scores to predict the survival of CRC patients (25). The differential FPR and significant survival differences were examined in the right- and left-sided CRC with IV stage (26). So, we speculated that chronic inflammation might be involved in survival differences of the right- and left-sided diseases. Here, we focused on evaluating the possible role of tumor primary location and preoperative FPR in the prognosis of 1,181 radically resected patients with stage I–III CRC. We further investigated the effect of FPR on adjuvant chemotherapy response and RFS to understand the prognostic difference in these patients.

## Materials and Methods

### Population

The study was undertaken at the Second Affiliated Hospital of Nanchang University (Nanchang, China) from January 2013 to August 2016 and was approved by the Medical Ethics Committee of the hospital. The eligible participants should fit for the following inclusion criteria: (1) newly diagnosed stage I–III CRC patients by clinical characteristics, imaging, clinical laboratory, and pathological examination; (2) agreement to participate and sign an informed consent form; (3) radical resection with histologically negative resection margins; (4) agreement to provide data of clinical characteristics, contact information, and peripheral blood sample. On the contrary, the exclusion criteria for each participant were as follows: (1) patients received any treatment such as neoadjuvant therapy, anticoagulant therapy, or long-term use of non-steroidal anti-inflammatory drugs in recent 3 months before diagnosis; (2) patients were combined with other malignancies, recent bacterium, or virus infection as well as trauma; (3) patients suffered from liver, blood, kidney and autoimmune disease, enterobiasis or cardio-cerebrovascular disease such as stroke, atherosclerosis, coronary heart disease, cardiac infarction, as well as vein thrombosis.

The eligible patients were stratified into the left- and right-sided subgroups according to tumor sidedness. The disease derived from the cecum, ascending colon, hepatic flexure, and transverse colon was recognized as right-sided CRC, whereas the disease originated from splenic flexure, descending colon, sigmoid colon, and rectum was considered as left-sided disease. The baseline characteristics, pathological detection, and surgical operation, as well as adjuvant chemotherapy after surgical operation, were gathered from the medical record. The chemotherapic regimens were classified as follows: single fluorouracil derivative oral anticancer agents such as Capecitabine or Tegafur, Gimeracil, and Oteracil potassium capsules [5-Fluorouracil (5-FU)]; Capecitabine combined with Oxaliplatin regimen (XELOX); combined 5-FU, Leucovorin Calcium, and oxaliplatin regimen (FOLFOX); and combined 5-FU, Leucovorin Calcium, and Irinotecan regimen (FOLFIRI). We obtained the survival data by 3 years' follow-up (3 months a time in the first 2 years, 6 months in the third year) and its deadline was August 30, 2019. The survival endpoints were RFS and OS. RFS was evaluated by standard radiologic criteria; it was measured from the date of surgical resection to documented first radiologic recurrence or distal metastasis and was censored at last follow-up. OS was defined as the time from the first diagnosis to death or the deadline.

### Laboratory Detection

To detect FPR, we collected peripheral blood specimens from each included patient from 7:00 to 10:00 am ahead 1 week of surgical operation. Two-milliliter serum and plasma specimens were obtained by 3,000 r/min centrifugation for 10 min. Plasma Fib was measured by Clauss method using SYSMEX CA-7000 machine (Sysmex, Tokyo, Japan). Immuno-turbidimetric assay was used to detect serum pre-Alb with machines of OLYMPUS 5400 (Beckman Coulter, Tokyo, Japan). The inter- and intra-batch coefficients of variation of these biomarkers were <5%. The ratio was calculated according to the following formula: FPR = (plasma Fib/serum pAlb) × 1000. We selected 18.3 as the optimal cutoff value of circulating FPR according to our previous study (24). Then, the patients were classified into high- and low-FPR subgroups according to the cutoff value. Both high- and low-FPR implied chronic high- and low-grade inflammation, respectively.

### Statistics

The baseline characteristics of the included patients were presented by number and proportion. Detected data with skewness distribution were shown with median and 25/75 percentile. RFS and OS emerged as median survival months. The Pearson χ^2^ test was used to compare the distribution differences of counting data in different groups. Mann–Whitney *U*-test was selected to detect the difference between two groups with skew distribution data. Kaplan–Meier curve with log-rank test and Cox regression analysis were selected to examine survival differences of the patients stratified by tumor location or FPR. Multivariate analysis was conducted by backward stepwise Cox regression modeling with covariates of the baseline and pathological characteristics, treatment, and other confounder factors. The strength between them was measured by hazard ratio (HR) and 95% confidence interval (CI). SPSS software v.17.0 (SPSS Inc., Chicago, USA) and GraphPad Prism 8.2.1 (GraphPad Software Inc., San Diego, USA) were used in all statistical analyses and *p* < 0.05 was defined as statistical significance in the present study.

## Results

**Figure 1:**
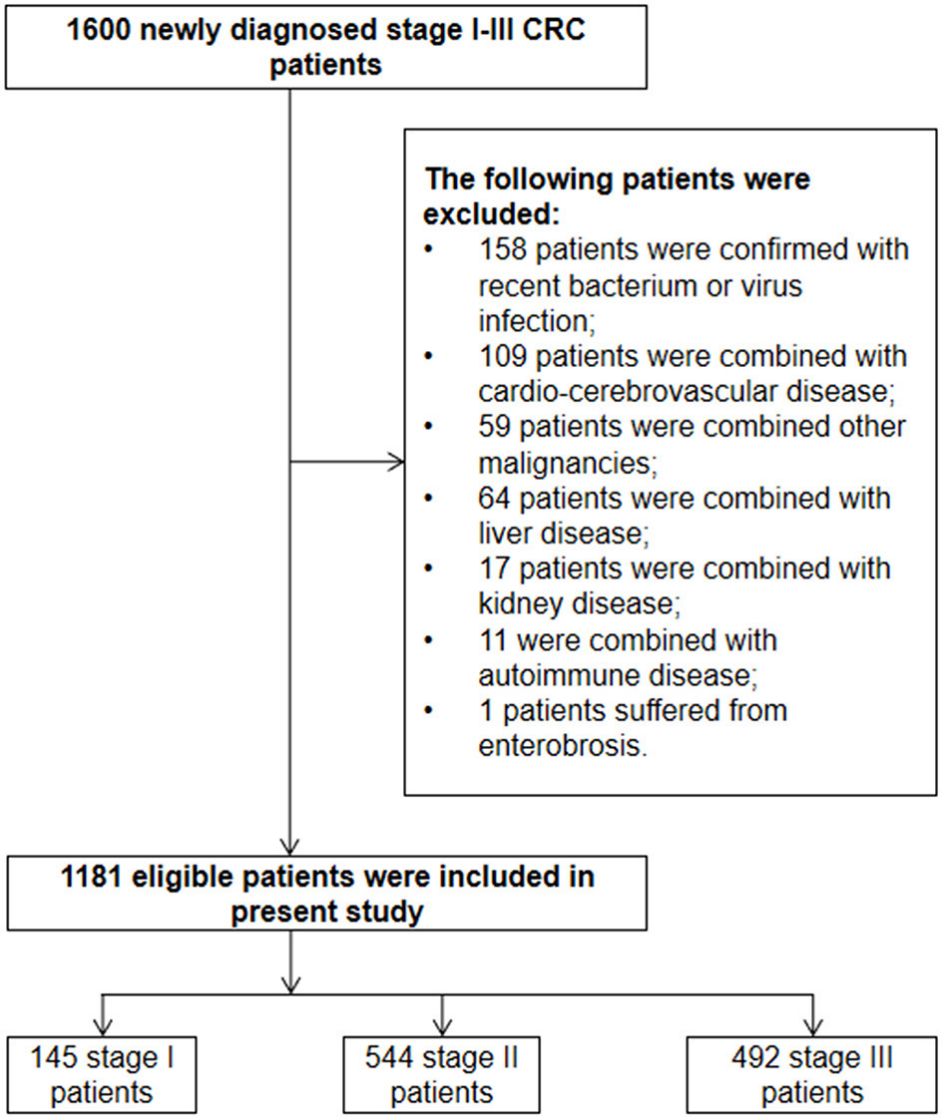
The detailed enrolled procedure of eligible patients in our study.

The detailed enrolled procedure is presented in Figure 1. A total of 1,600 newly diagnosed stage I–III CRC patients in the range of January 2013 to August 2016 were enrolled to identify the eligible cases according to the inclusion and exclusion criteria. As a result, 1,181 stage I–III CRC patients were finally enrolled in the present study. Despite the fact that primary rectal and distal colon cancer patients were subjected to different staging procedures and treatment approaches, no survival difference was observed between them (Supplementary Figures 1A–D). So, we considered two of them as CRC with left-tumor location. The demographic and pathological characteristics, as well as treatment data, are displayed in Table 1.

**Table 1:** The baseline characteristics of 1,181 stage I–III surgical colorectal cancer patients in the study.

**Characteristics**	**Right CRC (*N* = 283)**	**Left CRC (*N* = 898)**	***p*-value**
Gender (male), *N* (%)	162 (57.24)	548 (61.02)	0.26
Age (≥60 years), *N* (%)	82 (28.97)	225 (25.05)	0.19
Smoking (yes), *N* (%)	49 (17.31)	172 (19.15)	0.49
Drinking (yes), *N* (%)	34 (12.01)	125 (13.92)	0.41
Hypertension (yes), *N* (%)	53 (18.73)	167 (18.60)	0.96
Diabetes (yes), *N* (%)	31 (10.95)	112 (12.47)	0.50
**Invasion**, ***N*** **(%)**			
T1–3	47 (16.61)	261 (29.06)	<0.01
T4	236 (83.39)	637 (70.94)	
**Node metastasis**, ***N*** **(%)**			
N0	172 (60.78)	516 (57.46)	0.32
N1^*^	111 (39.22)	382 (42.54)	
**TNM stage**, ***N*** **(%)**			
I	18 (6.36)	127 (14.14)	<0.01
II	154 (54.42)	390 (43.43)	
III	111 (39.22)	381 (42.43)	
**Differentiation**, ***N*** **(%)**			
Moderate-well	242 (85.51)	791 (88.08)	0.28
Poor	35 (12.37)	83 (9.24)	
Unknown	6 (2.12)	24 (2.67)	
Chemotherapy (yes), *N* (%)	207 (73.14)	649 (72.27)	0.77

CRC, colorectal cancer; N1^*^, positive of node metastasis; the disease derived from the cecum, ascending colon, hepatic flexure, and transverse colon was recognized as right-sided CRC; the disease originated from splenic flexure, descending colon, sigmoid colon, and rectum was considered as the left-sided disease.

Eight hundred and ninety-eight and 283 patients were diagnosed as left- and right-sided CRC; 12.28, 46.06, and 41.66% of them were stage I (127 for left, 18 for right), stage II (390 for left, 154 for right), and stage III (381 for left, 111 for right) patients, respectively. Significant distribution differences of TNM (*p* < 0.01) and T (*p* < 0.01) stage were observed between the right- and left-sided cases. However, no distribution difference of other clinical characteristics was observed between the two subgroups.

**Figure 2:**
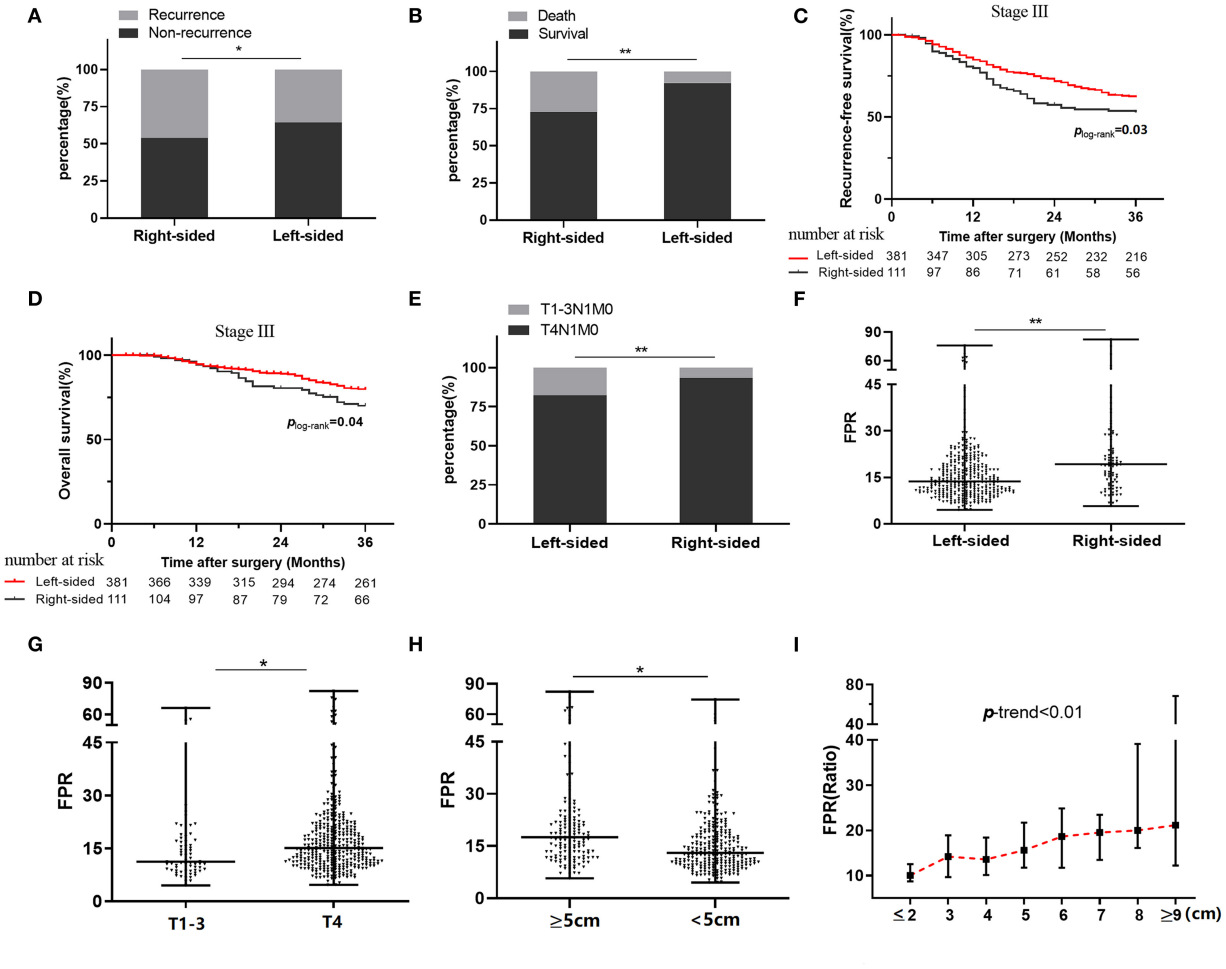
Relationship between clinical outcome, tumor sidedness, and preoperative FPR in stage III colorectal cancer patients. **(A)** Recurrence status in stage III patients stratified by tumor sidedness. **(B)** Death status in stage III patients stratified by tumor sidedness. **(C)** Kaplan–Meier curves for RFS in stage III patients with right-sided and left-sided cancer. **(D)** Kaplan–Meier curves for OS in stage III patients with right-sided and left-sided cancer. **(E)** Frequency distribution of T4 stage in left-sided and right-sided cancer. **(F)** Preoperative FPR in left-sided and right-sided cancer. **(G)** Preoperative FPR in T1–3 and T4 subgroup. **(H)** Preoperative FPR in subgroups with large (≥5 cm) or small (<5 cm) cancer size. **(I)** The change of median preoperative FPR in different cancer sizes. FPR, Fib to pre-Alb ratio; ^*^≤0.05. ^**^≤0.01.

The median follow-up period was 24 months (range, 3–36 months) after surgical resection, and 7.87% of them were lost to follow-up. Two hundred and eighty-nine cases were recurrent, 166 cases were dead in the follow-up period. Moreover, 8.28, 16.54, and 38.15% of stage I, II, and III patients experienced radiologic recurrence; the death rates within each stage were 3.45, 11.03, and 20.33%, respectively. No survival difference was observed in the subgroups stratified by clinical characteristics and therapeutic regimens (Table 2). We also did not observe survival differences between the right- and left-sided patients in overall (*p* = 0.10 for RFS and *p* = 0.184 for OS), stage I (*p* = 0.88 for RFS and *p* = 0.97 for OS), and stage II (*p* = 0.90 for RFS and *p* = 0.76 for OS) subgroup (Table 2), whereas recurrence rate (45.95 vs. 38.01%, *p* = 0.05) and mortality (27.02 vs. 18.37%, *p* < 0.01) of the right-sided individuals with stage III disease were significantly higher than the left-sided patients (Figures 2A,B). Moreover, survival outcome (*p*_log−rank_ = 0.03, adjusted HR = 1.41, and 95% CI = 1.02–1.94 for RFS; *p*_log−rank_ = 0.04, adjusted HR = 1.55, and 95% CI = 1.01–2.38 for OS) within the stage III right-sided CRC patients were significantly worse than the left-sided cases (Figures 2C,D and Table 2).

**Table 2:** Cox analysis of common clinical characteristics and primary tumor location within 1,181 surgical patients with stage I–III CRC and subgroups stratified by TNM stage in the study.

		**RFS**				**OS**			
**Population**	**Comparison**	**Univariate**		Multivariate^*^		**Univariate**		Multivariate^*^	
		***p*-value**	**HR (95% CI)**	***p*-value**	**HR (95% CI)**	***p*-value**	**HR (95% CI)**	***p*-value**	**HR (95% CI)**
Stage I–III	Gender (Male)	0.75	0.96 (0.76–1.22)	0.81	0.97 (0.75–1.25)	0.68	1.07 (0.78–1.46)	0.75	0.95 (0.69–1.31)
	Age (≥60 years)	0.20	1.18 (0.92–1.52)	0.26	1.16 (0.90–1.49)	<0.01	1.55 (1.12–2.13)	<0.01	1.57 (1.36–8.41)
	Smoking (yes)	0.69	0.94 (0.70–1.27)	0.71	1.07 (0.75–1.54)	0.38	0.83 (0.55–1.25)	0.86	0.95 (0.57–1.60)
	Drinking (yes)	0.64	0.92 (0.65–1.30)	0.83	0.96 (0.68–1.37)	0.45	0.83 (0.52–1.34)	0.51	0.85 (0.53–1.37)
	Hypertension (yes)	0.51	0.90 (0.67–1.22)	0.44	0.89 (0.65–1.21)	0.28	1.22 (0.85–1.76)	0.50	1.14 (0.78–1.66)
	Diabetes (yes)	0.55	1.11 (0.79–1.56)	0.63	0.92 (0.65–1.30)	0.31	1.25 (0.81–1.93)	0.91	1.03 (0.66–1.60)
	Chemotherapy (yes)	<0.01	1.55 (1.17–2.05)	0.80	0.96 (0.71–1.31)	0.63	1.09 (0.77–1.53)	0.11	0.73 (0.50–1.07)
	Right vs. Left^#^	0.13	1.23 (0.95–1.59)	0.11	1.24 (0.96–1.61)	0.12	1.31 (0.93–1.84)	0.18	1.26 (0.90–1.77)
Stage I	Right vs. Left^#^	0.63	1.45 (0.32–6.62)	0.88	1.13 (0.24–5.40)	0.59	1.83 (0.21–16.41)	0.97	0.96 (0.07–12.75)
Stage II	Right vs. Left^#^	0.95	0.99 (0.62–1.56)	0.90	0.97 (0.61–1.55)	0.81	0.93 (0.53–1.65)	0.76	0.91 (0.51–1.64)
Stage III	Right vs. Left^#^	0.04	1.40 (1.01–1.93)	0.04	1.41 (1.02–1.94)	0.04	1.55 (1.01–2.38)	0.04	1.55 (1.01–2.38)

*RFS, recurrence-free survival; OS, overall survival; HR, hazard ratio; CI, confidence interval; p-value, p value of Cox regression; left^#^, the left-sided disease included distal colon cancer and rectal cancer*;

* *the results were adjusted by age, gender, smoking, drinking, diabetes, hypertension, chemotherapy, T, N, differentiation, cancer size*.

In stage III patients, the frequency distribution of the T4 stage within the left-sided cases was significantly lower than that it in the right-sided patients (*p* < 0.01) (Figure 2E). Preoperative FPR was higher in subgroups with the right-sided disease (*p* < 0.05), T4 stage (*p* < 0.05), and cancer bulk ≥5 cm (*p* < 0.05) compared to the counterparts (Figures 2F–H). Moreover, circulating FPR was gradually rising in the patient with increased cancer bulk (*p* trend < 0.01) (Figure 2I).

**Figure 3:**
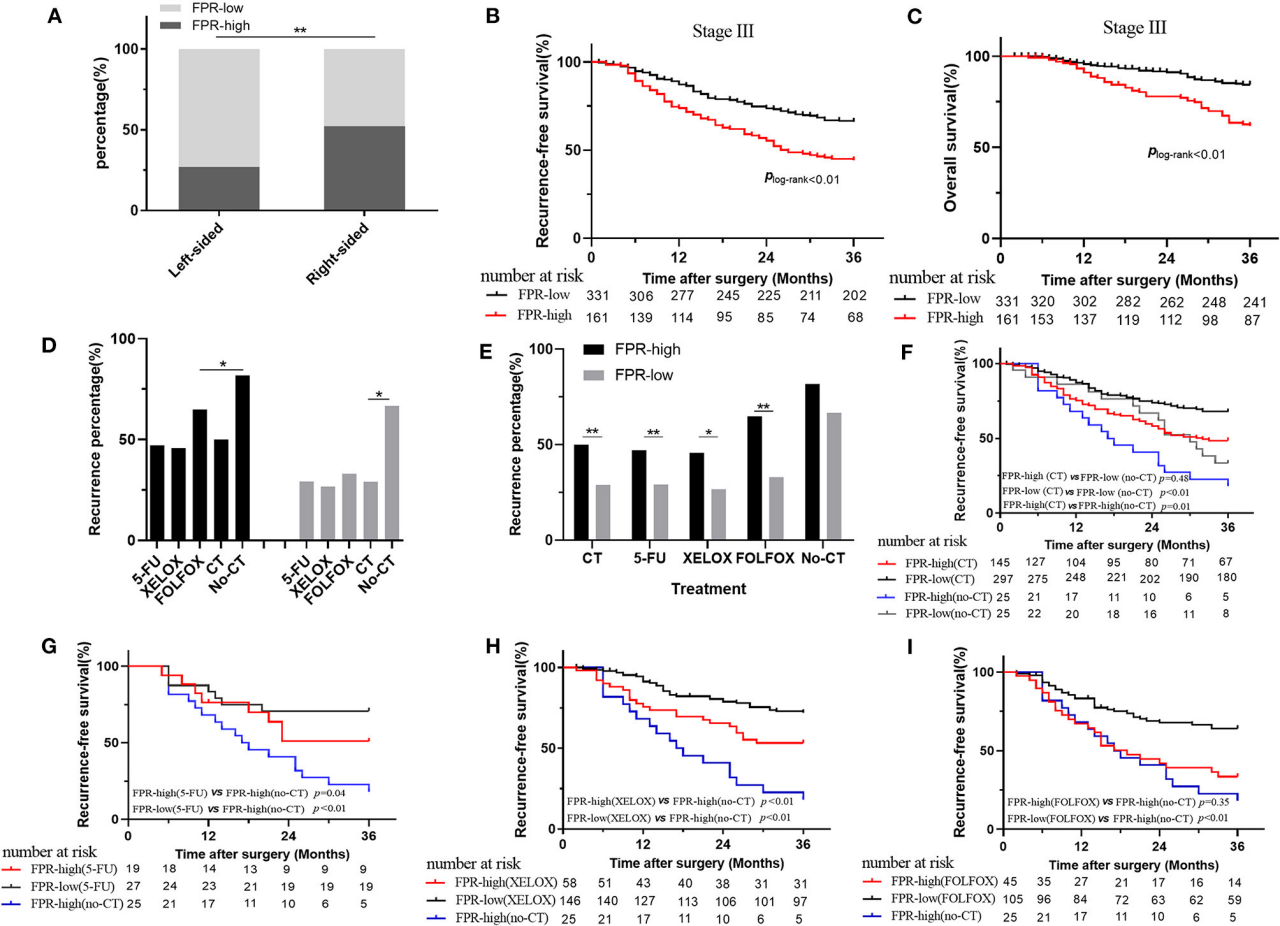
Association of preoperative FPR with clinical outcome in stage III colorectal cancer patients. **(A)** The distribution of high- and low-FPR in left-sided and right-sided cancer. **(B)** Kaplan–Meier curves for RFS in the stage III patients with high and low FPR. **(C)** Kaplan–Meier curves for OS in the stage III patients with high and low FPR. **(D,E)** The recurrence rate comparison in stage III patients with or without chemotherapy treatment. **(F)** Kaplan–Meier curves for RFS in high- and low-FPR chemotherapy-treated subgroups and non-chemotherapy-treated subgroup. **(G)** Kaplan–Meier curves for RFS in non-chemotherapy-treated patients, low- and high-FPR subgroups with the treatment of 5-FU. **(H)** Kaplan–Meier curves for RFS in non-chemotherapy-treated patients, low- and high-FPR subgroups with the treatment of XELOX. **(I)** Kaplan–Meier curves for RFS in non-chemotherapy-treated patients, low- and high-FPR subgroups with the treatment of FOLFOX; HR, hazard ratio; FPR, Fib to pre-Alb ratio; ^*^≤0.05; ^**^≤0.01.

In this study, a high-frequency distribution of evaluated FPR was also observed in the right-sided patients (*p* < 0.01 for 52.30 vs. 27.00%) (Figure 3A). Prognosis of the high-FPR patients was extremely inferior to the cases with low-FPR (*p*_log−rank_ < 0.01 for RFS, *p*_log−rank_ < 0.01 for OS) (Figures 3B,C), and the significant associations were observed when they were adjusted by confounders including clinical baseline and pathological characteristics, treatment, and primary tumor location (*p* < 0.01, adjusted HR = 1.96 and 95% CI = 1.42–2.70 for RFS; *p* < 0.01, adjusted HR = 2.44 and 95% CI = 1.459–3.75 for OS) (Table 3). However, there was no survival difference within the high- or low-FPR patients with different tumor locations (Supplementary Figures 1E,F).

**Table 3:** Cox analysis of preoperative FPR in 492 surgically resected stage III CRC patients.

		**Cox regression**					
**Variant**	**Outcome**	**Univariate**		**Multivariate**			
				**(****1****)**		**(****2****)**	
		***p*-value**	**HR (95% CI)**	***p*-value**	**HR (95% CI)**	***p*-value**	**HR (95% CI)**
FPR	RFS	<0.01	1.97 (1.46–2.67)	<0.01	1.96 (1.43–2.69)	<0.01	1.96 (1.42–2.70)
	OS	<0.01	2.76 (1.82–4.17)	<0.01	2.44 (1.59–3.75)	<0.01	2.44 (1.59–3.75)

*FPR, fibrinogen-to-prealbumin ratio; RFS, recurrence-free survival; OS, overall survival; HR, hazard ratio; CI, confidence interval; (1), was adjusted by gender, age, tobacco, alcohol, diabetes, hypertension, chemotherapy, radiotherapy, T, N, differentiation, cancer size; (2), was adjusted by gender, age, tobacco, alcohol, diabetes, hypertension, chemotherapy, radiotherapy, T, N, differentiation, cancer size, and primary tumor location*.

Due to high medical costs and poor physical conditions, 50 of the stage III CRC patients did not receive any adjuvant therapy after surgical resection. Four hundred and forty-two patients received adjuvant chemotherapy, among which 51 patients received adjuvant chemoradiotherapy. In the chemotherapy-treated patients, 46, 204, 150, and 5 of the stage III cases received the treatments of single 5-FU, XELOX, FOLFOX, and FOLFIRI, respectively. However, 37 patients received the treatment without a defined regimen. In chemotherapy-treated and the non-treated groups, high- and low-FPR patients harbored 48.74% and 29.48, 81.82, and 66.67% recurrence rates, respectively. The recurrence rate of adjuvant chemotherapy-treated patients was significantly lower than the patient without the treatment, regardless of FPR status (Figure 3D). The high-FPR patients harbored significantly high recurrence rate compared to the low FPR cases in chemotherapy-treated patients (*p* < 0.01 for 48.74 vs. 29.48%), especially in 5-FU (*p* < 0.01 for 47.10 vs. 29.20%), XELOX (*p* = 0.02 for 45.15 vs. 26.63%), and FOLFOX (*p* < 0.01 for 61.59 vs. 33.78%) subgroups (Figure 3E). However, no recurrence difference was observed in non-chemotherapy-treated high- and low-FPR patients (Figure 3E). Low-FPR patients harbored the best RFS with the treatment of chemotherapy, and the outcomes of chemotherapy-treated low- and high-FPR patients were superior to those without the treatment (Figure 3F and Supplementary Figure 1G). Low-FPR patients with treatment of 5-FU, XELOX, FOLFOX, and the high FPR patients without chemotherapy harbored the best and worst RFS. The survival of high-FPR patients without chemotherapy was inferior to the cases undergoing the treatment, especially in 5-FU- and XELOX-treated subgroup (Figures 3G–I). However, no RFS difference was observed in high- and low-FPR patients with adjuvant radiotherapy (Supplementary Figure 1H).

## Discussion

In this study, we investigated the role of tumor laterality and chronic inflammation in the survival outcome of stage I–III CRC individuals. We found that the prognosis of the right-sided stage III patients who harbored high T4 stage was worse than the right-sided cases. Preoperative high FPR was observed in stage III right-sided, T4 stage, and large cancer bulk subgroups, and it was significantly associated with poor prognosis of the patients regardless of tumor location. Moreover, the clinical outcome of high-FPR patients with treatment of adjuvant chemotherapy was inferior to the low-FPR cases with the same treatment, but was superior to the patients without chemotherapy after surgical resection.

Colorectal tissue within different locations is derived from different embryonic tissues (27). The distinct pathological and genetic features of the left- and right-sided CRC contribute to heterogeneous outcomes (28). In our study, the clinical outcome of the right-sided stage III patient was inferior to the left-sided cases, not the patients with the early-stage disease, indicating that tumor laterality was associated with prognosis in the stage III patients, and the right-sided cases were more likely to experience recurrence and metastasis after the radical operation. The result is consistent with the finding reported by Petrelli et al. (29). We also observed a significant difference in preoperative FPR and T4 patients' distribution in the left- and right-sided cases, the T4 patients harbored extremely high FPR, and preoperative FPR was found to be positively associated with cancer size. These findings demonstrated that the disease triggered level of chronic inflammation mainly relying on cancer bulk, and the right-sided patients with T4 stage and large cancer size harbored chronic high-grade inflammation compared to the left-sided cases. Moreover, no survival difference between the right- and left-sided patients was observed in subgroups stratified by FPR, suggesting that FPR was related to the poor survival of patients regardless of primary tumor location. High FPR was still significantly associated with poor prognosis of the cases when it was adjusted by other confounders including tumor laterality, revealing that chronic high-grade inflammation was an independent prognostic factor for the disease. Our previous study also indicated that elevated neutrophil-to-lymphocyte ratio (NLR) was significantly associated with diminished RFS, OS, and cancer-specific survival in surgical patient with stage I–III CRC. These findings indicated that common inflammatory ratios, FPR and NLR, could be independent prognostic factors for the disease.

Our previous study showed radio-chemotherapy resistance in right-sided metastatic CRC patients (26). In our study, we found that low FPR patients harbored the lowest recurrence rate and best RFS with the treatment of adjuvant chemotherapy, and the non-recurrence rate and survival of high FPR patients with the same treatment were superior to the patients without any adjuvant chemotherapy. These findings illustrated that survival benefit magnitude from adjuvant chemotherapy differed by the grade of chronic inflammation, low-grade inflammation could benefit more from adjuvant chemotherapy, while chronic high-grade inflammation might weaken sensitivity to common adjuvant chemotherapy, leading to heterogeneous clinical chemotherapy response and outcome in stage III surgical cases.

It is well-known that the interaction of genetic and environmental factors contributes to the onset and metastasis of CRC (30). Distinct genetic features might be one cause leading to survival differences in stage III patients with various tumor locations. Deficient DNA mismatch repair (MMR) proteins could impair genomic stability, leading to tumorigenesis, chemoresistance, and progression of the disease (31–34). Consensus molecular subtype (CMS) 1 and 3 with hypermutation of *KRAS, BRAF, PIK3CA*, and MMR pathway gene are commonly observed in the right-sided disease, while the left-sided disease usually harbors mediate and high copy number variation (35). Moreover, microsatellite-stable patients have been reported to benefit more from fluorouracil-based adjuvant chemotherapy than the cases with microsatellite instability (36, 37). On the other hand, rich expression of IFN-γ, CXCL9, and CXCL10 and significantly high CTLA-4 or PD-1^+^ CD8/CD4 T cells were observed in the tumor microenvironment of CMS 1 patients (35). An increasing gut microbial richness was also reported from the right-sided colon to the left-sided disease (38). *Escherichia-Shigella* and *Prevotella*, which were highly enriched in the right-sided CRC, appeared to be linked to elevated IL-17-producing cells and up-regulation of STAT3 and IL-6 in the mucosa of CRC patients (38, 39). Elevated IL-6 produced from the cancer microenvironment could effectively inhibit the synthesis of Alb and pre-Alb (40), whereas IL-17 facilitated cisplatin resistance in CRC by inhibiting cancer cell apoptosis through targeting p-Akt, Bax, Bcl-2, and mTOR (41), promoting its pro-proliferative and antiapoptotic properties (42). Moreover, hyperfibrinogenemia is a common clinical phenomenon in CRC patients, especially in the advanced disease. Fib has been reported to function as a scaffold to promote cancer progression (43). Thus, FPR implied chronic high-grade inflammation triggered by CRC cell, infiltrated gut commensal microorganisms, and immune cells as well as stromal cells conferred to impaired chemosensitivity, resulting in poor response to adjuvant chemotherapy and unsatisfactory survival in the right-sided cases.

In this study, we confirmed that high-grade inflammation within the left- and right-sided stage III surgical patients impaired survival benefit from adjuvant chemotherapy, leading to poor survival of the stage III cases. We also found that primary tumor location might be just a confounder factor for the disease while high-grade chronic inflammation was an independent prognostic factor for the patients. Despite the interesting findings, several limitations should be addressed as follows. We categorized transverse colon cancer as a right-sided disease, which might influence our findings. We could not obtain the pathological sample from each enrolled patient. *RAS* and *BRAF*, as well as microsatellite instability status, were unavailable from each enrolled patient in our study. Hence, we did not investigate the role of *RAS* and *BRAF* status in the survival of patients with right- and left-sided diseases.

In summary, our study reveals that chronic high-grade inflammation confers impaired sensitivity to common adjuvant chemotherapy, leading to the poor outcome of the patients with stage III disease. Further study is warranted to validate our findings and to investigate the clinical utility of anti-inflammation therapeutic target antibody combined with adjuvant chemotherapy for the high-FPR patients.

## Data Availability Statement

The original contributions presented in the study are included in the article/Supplementary Material, further inquiries can be directed to the corresponding author/s.

## Ethics Statement

The studies involving human participants were reviewed and approved by the Medical Ethics Committee of the Second Affiliated Hospital of Nanchang University. The patients/participants provided their written informed consent to participate in this study.

## Author Contributions

H-QY provided the idea, established the study design, performed the statistics, and written the manuscript. X-HY screened and selected eligible patients, laboratory detection, and follow-up. Y-CL contributed to data preparation, performed, and verified the statistic results. FS contributed to sample collection, and manuscript preparation. X-XC provided the idea and research funding for this study and revised and approved this study.

## Conflict of Interest

The authors declare that the research was conducted in the absence of any commercial or financial relationships that could be construed as a potential conflict of interest.

## Supplementary Material

Supplementary Figure 1 Survival comparison in overall or stage III population. **(A)** Kaplan–Meier curves for Recurrence-free survival (RFS) in stage I-III patients with rectal and left-sided (excluded rectum) cancer; **(B)** Kaplan–Meier curves for overall survival (OS) in stage I-III patients with rectal and left-sided (excluded rectum) cancer; **(C)** Kaplan–Meier curves for RFS in stage III patients with rectal and left-sided (excluded rectum) cancer; **(D)** Kaplan–Meier curves for OS in stage III patients with rectal and left-sided (excluded rectum) cancer; **(E)** Kaplan–Meier curves for RFS in FPR-high or FPR-low patients with right-sided or left-sided stage III cancer; **(F)** Kaplan–Meier curves for OS in FPR-high or FPR-low patients with right-sided or left-sided stage III cancer; **(G)** Kaplan–Meier curves for RFS in non-chemotherapy treated patients, low FPR chemotherapy-treated cases and first-line 5-FU, XELOX, FOLFOX treated high FPR subgroups; **(H)** Kaplan–Meier curves for RFS in FPR-high or FPR-low patients receiving radiotherapy. FPR, Fib to pre-Alb ratio; CT, chemotherapy; 5-FU, 5-fluorouracil; XELOX, capecitabine, leucovorin, oxaliplatin; FOLFOX, fluorouracil, leucovorin, oxaliplatin; ^*^≤0.05; ^**^≤0.01.
