# Supplementary figures and images for: High-Grade Inflammation Attenuates Chemosensitivity and Confers to Poor Survival of Surgical Stage III CRC Patients
Source: Front Oncol. 2021 Apr 23;11:580455. doi: 10.3389/fonc.2021.580455 (PMC8103203; doi:10.3389/fonc.2021.580455)

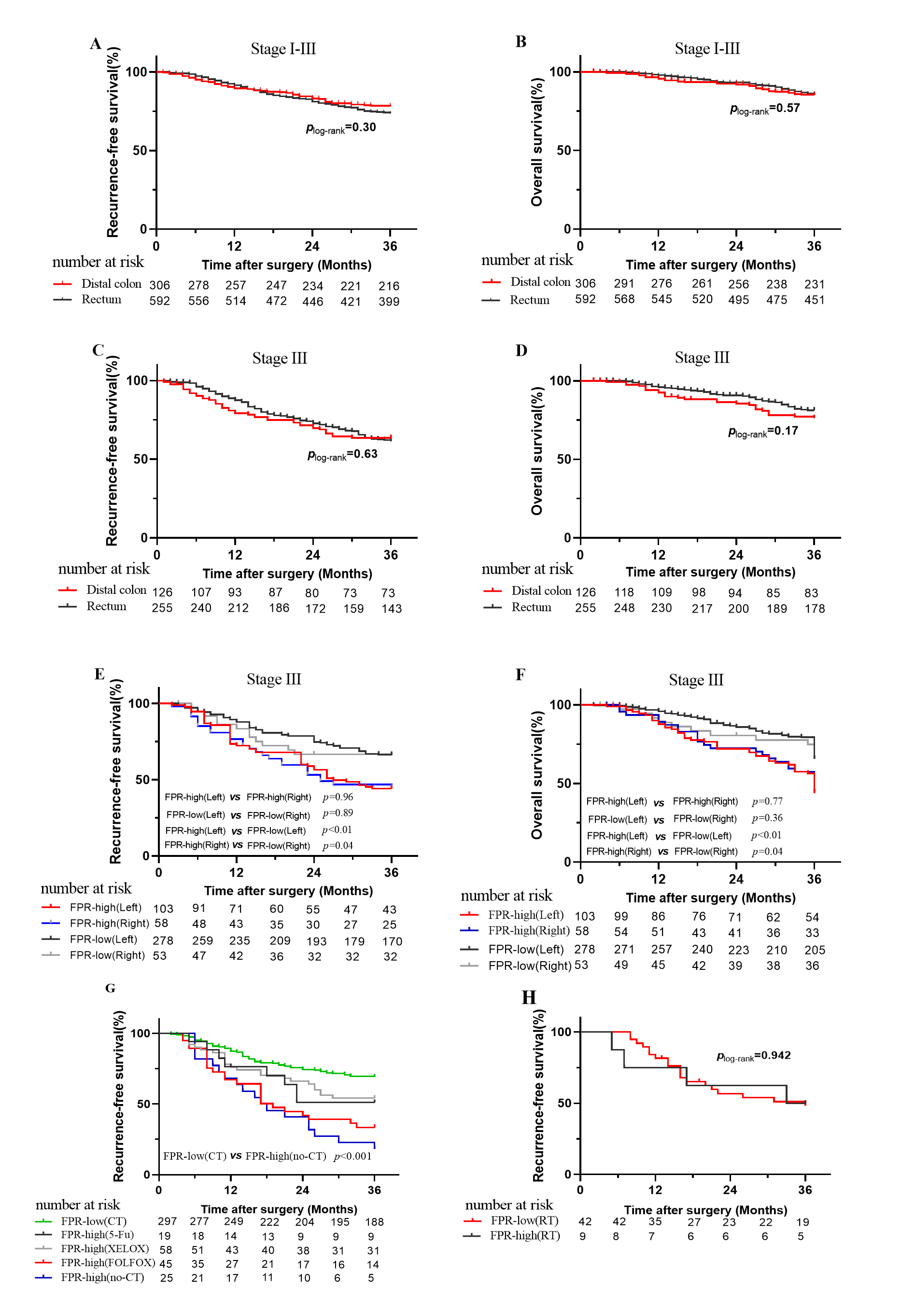

Supplement: Supplementary Figure 1 — Survival comparison in overall or stage III population. (A) Kaplan–Meier curves for Recurrence-free survival (RFS) in stage I-III patients with rectal and left-sided (excluded rectum) cancer; (B) Kaplan–Meier curves for overall survival (OS) in stage I-III patients with rectal and left-sided (excluded rectum) cancer; (C) Kaplan–Meier curves for RFS in stage III patients with rectal and left-sided (excluded rectum) cancer; (D) Kaplan–Meier curves for OS in stage III patients with rectal and left-sided (excluded rectum) cancer; (E) Kaplan–Meier curves for RFS in FPR-high or FPR-low patients with right-sided or left-sided stage III cancer; (F) Kaplan–Meier curves for OS in FPR-high or FPR-low patients with right-sided or left-sided stage III cancer; (G) Kaplan–Meier curves for RFS in non-chemotherapy treated patients, low FPR chemotherapy-treated cases and first-line 5-FU, XELOX, FOLFOX treated high FPR subgroups; (H) Kaplan–Meier curves for RFS in FPR-high or FPR-low patients receiving radiotherapy. FPR, Fib to pre-Alb ratio; CT, chemotherapy; 5-FU, 5-fluorouracil; XELOX, capecitabine, leucovorin, oxaliplatin; FOLFOX, fluorouracil, leucovorin, oxaliplatin; *≤0.05; **≤0.01. [file Image_1.TIF]
